# A Hybrid Feature Selection and Extraction Methods for Sleep Apnea Detection Using Bio-Signals

**DOI:** 10.3390/s20154323

**Published:** 2020-08-03

**Authors:** Xilin Li, Sai Ho Ling, Steven Su

**Affiliations:** School of Biomedical Engineering, Faculty of Engineering and Information Technology (FEIT), University of Technology Sydney (UTS), Sydney, NSW 2007, Australia; Xilin.Li@student.uts.edu.au (X.L.); Steven.Su@uts.edu.au (S.S.)

**Keywords:** feature extraction, feature selection, polysomnography, sleep apnea

## Abstract

People with sleep apnea (SA) are at increased risk of having stroke and cardiovascular diseases. Polysomnography (PSG) is used to detect SA. This paper conducts feature selection from PSG signals and uses a support vector machine (SVM) to detect SA. To analyze SA, the Physionet Apnea Database was used to obtain various features. Electrocardiography (ECG), oxygen saturation (SaO_2_), airflow, abdominal, and thoracic signals were used to provide various frequency-, time-domain and non-linear features (*n* = 87). To analyse the significance of these features, firstly, two evaluation measures, the rank-sum method and the analysis of variance (ANOVA) were used to evaluate the significance of the features. These features were then classified according to their significance. Finally, different class feature sets were presented as inputs for an SVM classifier to detect the onset of SA. The hill-climbing feature selection algorithm and the *k*-fold cross-validation method were applied to evaluate each classification performance. Through the experiments, we discovered that the best feature set (including the top-five significant features) obtained the best classification performance. Furthermore, we plotted receiver operating characteristic (ROC) curves to examine the performance of the SVM, and the results showed the SVM with Linear kernel (regularization parameter = 1) outperformed other classifiers (area under curve = 95.23%, sensitivity = 94.29%, specificity = 96.17%). The results confirm that feature subsets based on multiple bio-signals have the potential to identify patients with SA. The use of a smaller subset avoids dimensionality problems and reduces the computational load.

## 1. Introduction

During sleep, three basic respiratory disturbances are found. In these types, the most common disorder is sleep apnea (SA). The closure of the upper airway is repeated and temporary, and it is defined as SA in adults. SA can cause a complete breathing cessation, and the cessation lasts more than 10 s [1]. As a consequence, the sleep of people with SA is fragmented, and SA reduces the refreshing effects of sleep [2]. SA is associated with hypertension and cardiovascular diseases. It can also cause daytime sleepiness that has secondary risks such as vehicular accidents. SA should be objectively assessed to treat it. The apnea/hypopnea index (AHI) can evaluate SA. During sleep, it is considered to be the number of apnea and hypopnea events per hour. If it is greater than five or the minimum SaO_2_ value is less than 85% in adults, an AHI is defined as abnormal [3]. In general, in healthy people the AHI is less than 5. Five to fifteen is considered mild SA, and 15–30 is considered moderate SA, and greater than 30 is considered severe SA.

To diagnose SA, overnight polysomnography (PSG) is considered the gold standard. PSG monitors multiple bodily signals and records the comprehensive biophysiological changes during sleep. These biophysiological recordings include oxygen saturation (SaO_2_), midsagittal jaw movement, breath airflow, respiratory events, body position, snoring, electromyography (EMG), electroencephalography (EEG), electrocardiography (ECG), and electrooculography (EOG) [4]. A clinician must characterize pathological events and identify different parameters that are shown on the PSG to perform the diagnosis. The amount of data obtained is huge since the PSG records the changes in the whole night. As a result, for physicians, it is time-consuming to identify SA. Therefore, automatic detection is recommended. The aims of the automatic SA detection are to analyse the information from the PSG and diagnose SA automatically [2].

For SA detection, some studies have proposed automated methods using PSG signals. In general, SA is correlated with desaturation. Some methods utilize SaO_2_ and airflow signals for SA detection. Time-domain features are based on the number and intensity of oxygen desaturation events, and frequency-domain features are determined based on the intensity in the desaturation frequency range. In Reference [5], 17 features were used, and three classifiers (support vector machine (SVM), decision tree and probabilistic neural networks) were trained as a novel hierarchical classification method of diagnosing non-, mild, moderate and severe SA. Lempel-Ziv complexity, the central tendency measure, and the approximate entropy were used to extract features from SaO_2_ signals, and these features were used as the input into a multilayer perceptron network to provide a diagnosis [6]. A study [7] demonstrated the correlation between the drop and apnea events in the SaO_2_ signal. An algorithm was formulated, following the morphological criteria based on a physician, and a multivariate fuzzy temporal profile model was used to diagnose SA. The results of this experiment showed that the C4.5 Decision Tree was the best classifier for SA diagnosis. Gunes et al. [8] applied four features, that is, apnea and hypopnea indexes, arousal, the minimum SaO_2_ value during the rapid eye movement stage, and the ratio of the rapid eye movement stage time to sleep time. The multi-layer perceptron artificial neural networks (ANN) was used to detect SA. Airflow signals reflect respiration and its changes. In Reference [2], sudden changes were found in apnea fragments of airflow signals, and the amount of air was defined that the patient inhaled and exhaled, and a fuzzy set was used for diagnosis. Thoracic and abdominal signals are correlated with the airflow signal, and some studies focused on classifying SA using these two effort signals. A study [9] classified SA according to abdominal and thoracic signals. The discrete wavelet transformation was used to extract spectral components of the two effort signals, and the mean energy levels obtained were used as the input into a multi-layer neural network. This NN was able to classify SA, central SA, and mixed SA. An automated system for monitoring SA was introduced in Reference [10]. In this system, the spectral components obtained were the inputs for an ANN, and these components were extracted from the abdominal respiratory signals by the discrete wavelet transformation.

It was found some changes in EEG, ECG and chin EMG are correlated with sleep respiratory disturbances. A methodology for SA detection was presented based on frequency-domain methods in Reference [11]. In this methodology, features from ECG signals (in 1-min segments) were extracted by a heuristic splitting method and a relevance approach, and then a *k*-nearest neighbour algorithm classified SA according to these features. Hassan et al. [12] determined differences in the statistics, and spectral features were taken from apneic and no-apneic conditions in ECG signals. They separated the ECG signals into 1-minute segments, and then analysed the features: mean, variance, kurtosis, spectral flatness, centroid, spread, decrease, and slope. According to these features, the Bootstrap aggregation detected normal and apneic data. Three methods, that is, the least-squares SVM, the fast-Fourier transform, and the discrete wavelet transform, were used to develop a system to automatically recognise patients with SA [13]. Reference [14] applied the correlation dimension analysis, the detrended fluctuation analysis, three large Lyapunov exponents, and the spectral entropy were used to obtain nonlinear features in heart rate variability (HRV) signals. These features were inputs into an SVM to detect SA. The authors of Reference [15] modelled the Cepstral power using a hidden Markov model and applied it in the combination with an SVM to detect SA based on ECG signals.

EEG can provide useful sleep respiratory disturbance information. It has become one of the most significant signals for the diagnosis of sleep respiratory disturbance [16]. Trend features in Reference [17] were obtained by the Hilbert-Huang transformation and the Band-pass filtering. These features and the duration of SA were used to confirm SA. In Reference [18], to diagnosis SA, an adaptive neuro-fuzzy inference system was utilized. Features were obtained by the discrete wavelet transformation, and the system classified the apnea and the normal events in EEG signals. The Bispectral analysis was utilized to obtain bispectral characteristics called the quadratic phase coupling, and an ANN was used as a classifier to detect SA using EEG features [19]. The discrete wavelet transformation was employed to decompose the raw EEG signals. The GreyART network is one of the non-supervised machine learning systems, and the features from the wavelet transformation were used as the input for GreyART to identify SA [20]. The results of Reference [21] showed that features extracted by the autoregressive and the classification-by-least-squares SVM were able to detect changes in the EEG.

Many other SA diagnostic methods based on multiple bio-signals have been proposed. Al-Angari et al. [22] showed that the feature extraction from abdominal and thoracic respiratory effort signals, SaO_2_, and ECG signals could be applied with an SVM to detect apnea events. Two effort signals were used to compute the phase-locking value. Features were computed, including the standard deviation and the mean of the time between beats (RR intervals), the normalized and absolute powers in the high-frequency band, the low-frequency and the very-low-frequency bands, and the power ratio of the low-frequency band to the high-frequency band. Standard deviations and means of SaO_2_ were also computed. For classification, SVM classifiers were used with these features. A benchmark method for automatic detection SA was established based on ECG, airflow and SaO_2_ in Reference [23]. Each signal was decomposed by the wavelet transformation and the depth was 14. At every detail level, the mean, variance, and energy were computed. An ANN and other classifiers were compared and features from different signals were the inputs fed into classifiers. The best model was an ANN applied airflow signals. In addition, in SaO_2_ and ECG signals, features were used to train a linear discriminant model in Reference [24].

In automatic detection, feature extraction and selection play significant roles. Feature extraction is applied to obtain significant features that reflect bio-physiological criteria. Features with bio-physiological criteria can provide good classification performance. Feature selection can also reduce computational load. In the present study, the main contribution is to take the next step in the SA feature selection. We explore the importance of each feature extracted from PSG signals. In the evaluation of the importance of these features, the analysis of variance (ANOVA) and the rank-sum method are applied. Then, an SVM classifier is designed using the previously extracted features as the inputs. Feature extraction and selection pinpoint the best features for detecting SA since the selected features are with the best discrimination. In this study, the aim is to select features with the best discrimination, and they can be extracted from PSG signals and used to detect SA. The next section is about the feature extraction from different signals.

## 2. Materials and Methods

### 2.1. Sleep Apnea Dataset

**Figure 1:**
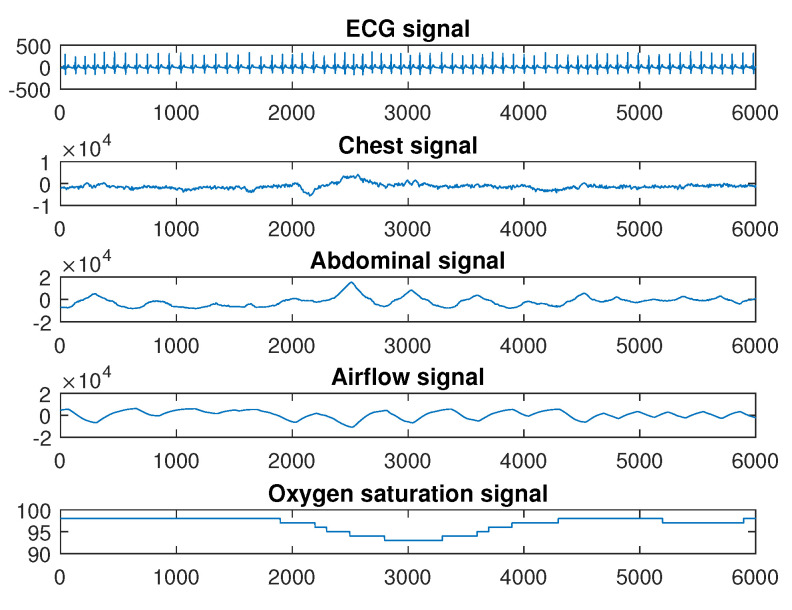
Example of one minute Polysomnography (PSG) signal.

The Physionet apnea dataset (https://physionet.org/content/apnea-ecg/1.0.0/) provided overnight PSG signals. In this study, the aim is to select multi-domain features with good discrimination from different kinds of bio-signals. In the Physionet apnea dataset, only 8 recordings (a01 through a04, b01, and c01 through c03) include five kinds of bio-signals (ECG signal, abdominal and thoracic signals, airflow signals, and oxygen saturation signals). Thus, these 8 recordings were used in this study. The dataset contained eight multi-signals with lengths of 455–529 min (mean ± std: 495 ± 21), and these recordings were sampled at 100 Hz. Each minute for SA in each signal was manually annotated by an expert. The subjects were 7 male and 1 female, and their ages were between 31 and 64 years (mean ± std: 43 ± 8). The heights of the subjects were 168–184 cm (mean ± std: 177 ± 6) and their weights were 63–121 kg (mean ± std: 88.5 ± 22). All information was recorded simultaneously, including ECG, thoracic, abdominal, airflow, and SaO_2_ signals. One minute of a PSG signal is shown as an example in Figure 1. Inductance plethysmography was used to obtain thoracic and abdominal signals, and airflow and SaO_2_ signals were measured using nasal thermistors and pulse oximetry, respectively. In each signal, one for each minute included the annotation, indicating apnea or normal event at that time. “A” indicates that apnea occurred during the following one-minute interval, while “N” indicates there was no apnea in the annotation. A total of 87 features were obtained from the abdominal and thoracic effort, airflow and SaO_2_, and ECG signals.

### 2.2. Feature Extraction

We extracted 87 features from ECG, SaO_2_, airflow, abdominal, and thoracic signals, obtained by time-, frequency-domain and non-linear methods in the work.

#### 2.2.1. Feature Extraction Using ECG Signal

In the part, we obtained time-domain, frequency-domain, and non-linear features. Table 1 shows ECG features 1 to 62. Preprocessing: We utilized a band-pass filter with 0.05–40 Hz to filter noise and artefacts and conduct the base-line correction. The filter was a 3rd-order infinite impulse response Butterworth. After de-noising, in the filtered ECG signal, the R-peaks were identified using the modified Pan-Tompkin algorithm. Here, a symmetric window of 120 ms was used to obtain all QRS (Q wave, R wave, and S wave) around the R-peaks. The heart rate, known as the RR interval, was considered as the time difference of consecutive R peaks. Due to the poor signal quality, a pre-processing step was done to obtain a set of RR intervals with more physiologically reasonable, taken as the HRV. In this paper, we employed the correction method from [25]. Besides the HRV and QRS complex, ECG-derived respiration (EDR) signals were also derived using the EDR method of Physionet. These signals reflect respiratory activities between the electrodes and the heart since the electrodes were placed on the patient’s thorax, and the electrical impedance changed during breathing. The motion of the thoracic cavity also influences the ECG signals [26]. Previous studies have stated that the heart rates of SA patients are lower than the heart rates of healthy people [27].

Time-domain features: Time-based features included (1) the root mean square consecutive difference using a set of R-peak amplitude (RMSSD_R_amp), (2) the number of pairs of adjacent HRV that the latter HRV parameter exceeded the former HRV parameter by greater than 50 ms (NN50_RR), (3) the standard deviation of the differences of consecutive HRV signals (SDSD_RR), (4) the standard deviation between the standard deviation of the HRV signal at the first 30 s and the standard deviation of the HRV signal at the second 30 s (tSD_RR), (5) the standard deviation of HRV signals (std_RR), (6) the mean, variance, kurtosis of ECG signals (mean, var, and kurtosis), (7) the mean of the RR interval (mean_RR), (8) the coefficient of the variance (the ratio of the standard deviation to the mean in EDR signals, CV_EDR), and (9) the mean of the R-peak amplitude (mean_R_amp).

Frequency-domain features: Features of the work were obtained by the wavelet transformation and the power spectral density in frequency-domain. The wavelet transformation has more advantages than classical Fourier transformation methods. The wavelet transformation uses a multi-scale basis. A varying window size is used to analyst non-stationary signals, and the size is narrow at high frequencies and broad at low frequencies. The functions of scaling and wavelet are applied by the wavelet transformation to decompose signals at approximate and detailed levels, and there is the correlation between high-pass and low-pass filters and these functions. The distribution of frequency power components is described by the power spectral density (PSD). The PSD method includes parametric methods and non-parametric methods. The advantage of parametric methods is higher accuracy, while the advantage of non-parametric is less computational complexity.

First, a level-9 wavelet transformation with Daubechies wavelets 4 were used to decompose EDR signals and obtained variances of the 9th and 2nd detail levels (var_EDR_D9 and var_EDR_D2). Second, a 3rd-order Symlet wavelet was the mother wavelet with a level number of 7 to decompose. The Shannon’s entropy (entropy_D1 to entropy_A7), the mean (mean_D1 to mean_A7) and the variance (var_D1 to var_A7) were extracted from one approximation and 7 detail coefficient levels, and the wavelet spectral density (WSD) was used to analyze RR intervals and the R amplitude (WSD_RR and WSD_R_amp). On the other hand, the PSD was applied to the RR intervals, and the dominant frequency was found in the 0.03–0.5 Hz frequency band (max_PSD_0.03/0.5_). We extracted the mean PSD in the 10–20 Hz and 80–100 Hz (mean_PSD_10/20_ and mean_PSD_80/100_). The PSD estimate was performed for the EDR signals and RR intervals and resulted in variances of 0.03–0.4 Hz (var_EDR_0.03/0.4_ and var_RR_0.03/0.4_). We utilized spectral flatness, centroid, spread, decrease, and slope.

Non-linear features: Recurrence is a fundamental characteristic of dynamic systems, and recurrence quantification analysis describes the structures of recurrence. In the study, we extracted three parameters, (1) V_MAX (the longest diagonal length), (2) DET (the percentage of recurrent points to use the minimal length recurrent point and to form diagonal lines), (3) LAM (in vertical lines of minimum length, the percentage of recurrent points). Similarly, in RR intervals, we extracted five serial correlation coefficients (SCrC_1_RR to SCrC_5_RR). Here, the QRS signal was processed by the principal component analysis (PCA) and the kernel principal component analysis (kPCA) [28]. These features correspond to the standard deviation of PCA and kPCA (std_PCA and std_kPCA), the maximum of the diagonal matrix of PCA and kPCA (max_dia_PCA and max_dia_kPCA), and the relative power in the second principal component (RP_2_PC).

#### 2.2.2. Feature Extraction Using Oxygen Saturation Signal

From the oxygen saturation signal, we obtained four features from the time- and frequency-domain. Features were those of Nos. 63–66 in Table 1. Preprocessing: There were many quantitative indices used to diagnose SA. The most widely used index in SaO_2_ includes the cumulative time less than a value, a 90% decline from baseline or the number of SaO_2_ less than a value, and a 3% decline from baseline. When the sample-to-sample differences were >8%, they were considered as non-physiological artefacts. To reduce non-physiological artefacts, the median value was calculated in the initial 10 s, and these artefacts were replaced by the median value. Here, data were down-sampled to 1 sample/s to be considered as low-amplitude artefacts.

Multi-domain features: For SaO_2_ recordings, the time- and frequency-domain features were obtained in this study. The feature set included the median of 60-second (med), and rapid restoration events identified as an increase of more than 4% in 10 s (RES4). Apnea events usually lasted 20–60 s. Therefore, the PSD obtained in desaturation events was correlated with the number and intensity of desaturation events. The PSD was calculated by a 5th-order Yule-Walker autoregressive estimate, and the mean in the 0.016–0.05 Hz frequency range was added to the feature set (mean_PSD_0.016/0.05_). Finally, a variable called the Poincare SD_1_ was computed, which indicates the short-term variability in the SaO_2_ signal [29].

#### 2.2.3. Feature Extraction Using Airflow Signal

Time- and frequency-domain features Nos. 67–75 (Table 1) were extracted from the airflow signal. Preprocessing: An apnea event in the airflow signal is also defined as the at least 10% decrease from its basal value, and this decrease lasts at least 10 s [7]. Apnea events are recorded when there are at least two missed breaths of length [30]. The airflow signal swings up during exhalation and down during inhalation, which is a direct time-domain change. We started the baseline correction using the median level of a window of 10 s. For noise removal, a 3rd-order Butterworth low-pass filter was used, and its cut-off frequency was 3 Hz. We down-sampled the airflow signal from 200 Hz to 1 Hz.

Multi-domain features: Apean indicates airflow decreases recurrently throughout the night, and this behaviour supported this study. Hence, the mean, median (med), and standard deviation (std) were extracted as time-domain features. On the other hand, the Welch method was applied to obtain PSDs since the airflow signal were non-stationary signals. A segment length of 5 samples with 2.5 overlapped samples was used. The means were calculated within 0–0.1 Hz and 0.4–0.5 Hz (mean_PSD_0/0.1_ and mean_PSD_0.4/0.5_) since there were at least two missed breaths of length which reduced the respiratory frequency, and then there was fast respiratory overexertion which increased the respiratory frequency. The wavelet transformation decomposed each airflow signal and extracted spectral differences between apnea and normal groups. The level-3 wavelet transformation with Daubechies wavelets = 3 was performed. Using the wavelet, a given signal was decomposed into one approximate coefficient and three detailed coefficients (mean_D1 to mean_A3).

#### 2.2.4. Feature Extraction Using Abdominal and Thoracic Signals

We used time- and frequency-domain methods to process abdominal and thoracic signals. Feature Nos. 76–81 were extracted from abdominal signals while Nos. 82–87 were extracted from thoracic events (Table 1). Preprocessing: A total closure of the upper airway is scored as the apnea. During sleep apnea events, the abdomen and thorax of patients move. For the work, we estimated the performance of abdominal and thoracic signals. First of all, we started the baseline correction using the median-level window of 10 s. We used a 3rd-order band-pass Butterworth filter in 0.05–40 Hz to remove noise and artefacts.

Multi-domain features: Firstly, abdominal signals were used to provide feature sets. The sum and standard deviation of the absolute value of each 60-s (sum_abs, std_abs), and the mean of every 60-s (mean) were put into the time-domain feature set. The Yule-Walker method was used to estimate the PSD, and the segment length was 40 samples. The PSD provided the mean in the 80–100 Hz (mean_PSD_80/100_). A wavelet of depth 2 was designed with Daubechies 2, and the mean of the 1st and 2nd detail levels were computed (mean_D1 and mean_D2). Then, the thoracic signal provided other features, which included the sum, standard deviation, median, variance, and mean of each 60-s (sum, std, med, std, mean, and var). The Yule-Walker method was also used and the mean in the 80–100 Hz frequency band was obtained (mean_PSD_80/100_).

### 2.3. Feature Selection

A total of features increase the training time of classifier, as time is wasted in processing redundant or potentially detrimental features, which limits real-time detection applications. Also, redundant features influence the high accuracy of the classification algorithm. Thus, before the classification stage, feature selection plays an important role. Feature selection can prevent overfitting of training and reducing computational load. A small subset of data is obtained by feature selection, and the subset is with high-discriminatory power, which maintains performance and shortens the training process time since feature selection leaves the most compact feature sets. For feature selection, which provides the most discriminating information between two classes, we employed a two-stage procedure: the statistical analysis and the SVM selection. In the first stage, according to the statistical performance of different features, redundant features are removed from the feature set, and other features are put into different classes [31,32,33,34]. Then, we evaluated the performance of different feature classes with a trained SVM model. In this case, the hill-climbing method and the *k*-fold cross-validation were used. The feature set was reduced, and the best feature class with good performance was selected via comparisons of sensitivity, specificity, and accuracy.

#### 2.3.1. Statistical Analysis

To select the features with good discrimination, we used two statistical tests: ANOVA and the rank-sum test. ANOVA [14,32] can analyse differences between group means and associated labels. The error (residual) sum of squares (*SSE*) was computed while the total sum of squares (*SST*) was calculated. We obtained the group sum of squares (*SSG*), group means, and the sum of squares errors by dividing by their respective degrees of freedom, $df_{group}$ and $df_{error}$. Resulting means were defined as the group mean squared and mean squared errors. Finally, we obtained the *F*-ratio. This ratio follows an $F_{v_{1},v_{2}}$ distribution, where *v*${}_{1}$ = $df_{group}$ and *v*_2_ = $df_{error}$, which is used to compute the *p*-value.

In the rank-sum test [34,35], the aim was to confirm that the two groups were independent. Two values were randomly selected from the first group and the second group, respectively, and these two values were compared. Let *n*_1_ and *n*_2_ be the numbers of the two samples, respectively. We ranked the combined *n*_1_ + *n*_2_ observations in ascending order and substitute a range of 1, 2... to the *n*_1_ + *n*_2_ observations. We assigned conflicting observations with their mean ranks. The *w_i_* (*i* = 1, 2) indicates the sum of the ranks corresponding to *n_i_* observations. Let *u* = min(*u*_1_,*u*_2_) and *u* will be compared with the desired critical value. The rank-sum test also obtains the *p*-value.

We used a simple threshold (ANOVA *p*-value < 0.05) and a simple number (*p*-value of the rank-sum test = 1) to classify the positive and negative features. The results of the ANOVA and the rank-sum test are presented in Table 1. In total, there were 87 features. Feature Nos. 1–62 were from each ECG minute, Nos. 63–66 were from SaO_2_ signals, Nos. 67–75 were from the airflow signal, Nos. 76–81 were from the abdominal signal, and Nos. 82–87 were from the thoracic signal. In Section 2.1, we mentioned eight PSG signals were used in this study. We found that all events of three PSG signals (c01 through c03) were labeled by normal, and there were no apnea events. Mentioned in the description of statistical analysis, both ANOVA and the rank-sum test used apnea and normal events in one patient to calculate *p*-value of each feature. Thus, in this statistical analysis stage, only five patients with two kinds of events were used, and eight patients’ PSG signals were used in the next SVM stage.

In this study, for each derived feature, statistical results from both apnea and normal events from the first patient were analyzed and a pair of *p*-values was obtained. This process was repeated until five patients’ PSG signals were used to provide five pairs of *p*-values for this feature. Then the next feature was taken by ANOVA and the rank-sum test. Each pair included one ANOVA *p*-value and another one of the rank-sum test. After deriving the *p*-values of the full feature set, features had the good discrimination between two groups and selected into the feature subset. We set λ_feature_ as the number of positive pairs for each feature. In this study, the maximum of λ_feature_ was equal to five since it was set to be the same as the number of patients. In Table 1, λ_feature_ of Feature No. 10 was 2, which means that two pairs of the Feature No. 10 were positive. Similarly, λ_feature_ of Feature No. 2 = 5, which means that this feature has been identified as a positive feature for all 5 patients. If the λ_feature_ of a feature was ≥ 1, as shown in Table 1, then this feature was put into the selected feature set and highlighted with an asterisk (*) in Table 1. Finally, 66 features were selected based on λ_feature_ being ≥ 1. The aim of this stage was to discard noisy and irrelevant features and reduce the computational load of the algorithm.

#### 2.3.2. SVM Selection

An SVM classifier was used in the second stage to find features with good discrimination. The SVM was introduced by Vapnik [36] and it is a classification method and regression analysis. It is a binary classifier and it constructs an optimal separating hyperplane (OSH) in an N-dimensional space. The input vector is transformed via nonlinear mapping into a K-dimensional space from the N-dimensional space.

A training data *D* = {(*x${}_{i}$*,*y${}_{i}$*)}${}_{i}^{L}$ constitutes of every training vector *x${}_{i}$*∈R${}^{n}$ and the matched label *y${}_{i}$*∈ (+1, −1). Every training vector *x${}_{i}$* is transformed via a nonlinear mapping function **z** = *⌀*(**x**) into a higher-dimension space F. Every vector **w**∈R${}^{n}$ is the normal vector to the hyperplane, and the points **z** lie on the hyperplane, and margin bias *b* is in R, and these parameters satisfy **w**·**z** + *b* = 0, while the input data satisfy the equation *y${}_{i}$*(**w**·**z** + *b*) − 1 ≥ 1 ∀*i*. The bias is computed by 2/‖**w**‖. When the OSH maximizes the bias to minimize $w^{2}$/2, the SVM constructs the optimal one. The quadratic programming with Lagrange multipliers α${}_{i}$ can solve a convex optimization problem and a dual problem.

Selecting a kernel function *K*(*x${}_{i}$*,*x${}_{j}$*) affects the performance of the SVM in the construction stage. In this study, we tested three kernels and selected the best one. Three kernels include Linear, Polynomial, and Radial basis function (RBF). To select the best performance of three kernel functions, we used three indexes, that is, accuracy, sensitivity, and specificity.

## 3. Results and Discussion

We classified all features into five groups (Classes A, B, C, D, and E) via the relationship between λ_feature_ and the number of PSG signal (*ν*_PSG_ = 5) from Table 1 and Table 2. In Table 1, the λ_feature_ of Feature No. 64 was 5 and was obtained from the SaO_2_ signals. The λ_feature_ of Feature No. 64 was equal to *ν*_PSG_ and was put into Class A. Similarly, the λ_feature_ of Feature No. 79 was 2 (*ν*_PSG_-3) and was from the abdominal signal. It was put into Class D. Three kernel functions, Linear, Polynomial and RBF affected the performance of the SVM. Considering all class features as inputs of the SVM, the performance of the kernel functions were evaluated using parameters. The parameters considered were Linear (*R*), Polynomial (*d*, and *R*) and RBF (σ and *R*). In these parameters, *R* is used in Linear, Polynomial and RBF. *R* is the regularization parameter, and *d* in Polynomial is the degree, and σ in RBF is the width.

It was found that eight PSG signals from the Physionet database were imbalanced since the number of normal events was much larger than that of apnea events, which affected sensitivity, specificity, and accuracy. The under-sampling balance method was used in this study. This method reduced the number of normal events and balanced the number of apnea events and the number of normal events in these eight PSG signals. The parameters, including *R*, *d*, σ, carried different values, that is, σ (1,5,25), *d* (2,3,4) and *R* (0.2,1,10). In order to detect the best classifier, the performance is presented by accuracy, sensitivity, and specificity via the *k*-fold cross-validation. We followed the hill-climbing algorithm mentioned, in order to detect the feature subset with the most discriminating information. Initially, Class A features were selected to feed into the SVM, and different kernels with different parameters were set. Then, the next class features were added, and this algorithm was repeated until all classes were added. The final feature subset was selected by comparing the performance. According to the results of the *k*-fold method and the hill-climbing algorithm, the means were computed and Table 3 shows the means (%) for accuracy, sensitivity, and specificity.

In Table 3, most of sets had good performance (more than 90% accuracy). However, the accuracy (≤90%) is highlighted in bold because these sets had worse performance than other sets. Compared the performance of different feature subsets, the results presented showed that Class A was better than the other classes.

To confirm the best feature subset, two statistical indexes, the standard deviation and the mean, were used to evaluate the results shown in Table 4. In this study, a lower feature class standard deviation indicates less fluctuation, and this feature class was more stable than other classes. A higher feature class mean indicates that the performance of this subset was better than those of other classes. From Table 4, it can be seen that the standard deviations of Class A were 4.27%, 1.82% and 2.79%, respectively. On the other hand, for mean values, Class A exhibited better performance. Its means of sensitivity, specificity and accuracy were 93.22%, 94.60%, and 93.93%, respectively. Comparing the classification performance results in Table 3 and Table 4, we find that the best feature subset was Class A (Nos. 2, 47, 64, 66, 77), which had better effectiveness and robustness.

Class A (No. 2, 47, 64, 66, 77) was the feature subset with the highest discriminating power. Features No. 2 and 47 were extracted from ECG signals, and Nos. 64 and 66 were extracted from SaO_2_ signals, and No. 77 was extracted from abdominal signals. During apnea events, patients with sleep apnea showed distinct patterns in the cardiac RR interval variation. Compared with the normal events, sleep apnea events increase the RR interval variation at lower heart rates and the decrease variation at faster rates. Feature No. 2 indicates the number of pairs of adjacent HRV samples that the latter HRV sample exceeded the former HRV sample by greater than 50 ms (NN50_RR). This feature indicates the different RR intervals of apnea and normal groups. On the other hand, lower heart rates lead to a change in the frequency domain. Feature No. 47 shows the spectral spread, and it is around the centroid and it reflects the standard deviation of the frequency range. The spectral spread shows that heart rates are lower in apneic parts, and the frequency values are lower than those in normal sub-bands. In apnea events, sudden downturns were followed by relatively fast recoveries in SaO_2_ values. Feature No. 64 describes rapid restoration events, an increase of more than 4% in 10 s (RES4), and shows rapid downturns and recoveries in oximetry values. Feature No. 66 is the Poincare SD_1_, which was obtained by comparing each SaO_2_ value against the previous one, and reflects the short-term variability. Sudden downturns and fast recoveries in SaO_2_ values affect this feature. The closure of the upper airway leads to SA. The patient’s abdomen is active during these closures. Feature No. 77 is the standard deviation of the absolute value of each minute of abdominal signals (std_abs). Their values are higher during apnea events than at the normality. We used absolute values since there was a fluctuation in abdominal signals that affected the standard deviation. Overall, selected features with the best discriminative power were associated with the bio-physiological criteria of SA.

The receiver operating characteristic (ROC) curve is related to the sensitivity and specificity of the classifiers. Ideally, the sensitivity value is 1, and the specificity value is 0. In this work, after determining that Class A was the best feature subset, the ROC area (AUC) was used to evaluate the different SVM models, and the *k*-fold method was also used. A comparison of AUCs is presented in Table 5. From Table 5, it found that the AUC of Linear (*R* = 1) was 95.23%, which held a better performance. From Table 3, it found that every accuracy in the Linear (*R* = 1) kernel was more than 90%, which means Linear (*R* = 1) was more stable than other kernels with parameters. Considering Class A features as the SVM input, the classification performance of the Linear (*R* = 1) kernel was 95.22%, while the sensitivity was 94.29%, and the specificity was 96.17%.

Five kinds of bio-signals (ECG, SaO_2_, airflow, abdominal, and thoracic signals) provided time-domain, frequency-domain, and non-linear features (show in Table 1), since these five signals are easy to collect, and they also show bio-patterns during apnea events are much different from patterns during normal events. Based on the λ_feature_ obtained by ANOVA and the rank-sum test, 87 kinds of features (shown in Table 2) were put into Classes A–E. The results of different classes were conducted by the statistical analysis shown in Table 3. This meant the features in Class A had the good discriminatory capability. The time- and frequency-domain features in Class A from ECG, SaO_2_, and abdominal signals were correlated with the bio-physiological criteria of apnea, such as the changed RR interval variation, sudden downturns and recoveries of SaO_2_ values, and the active abdomen muscles. SVM models were used to confirm the potential of these selected features. The SVM model with the Linear (*R* = 1) provided the high performance (the accuracy of 95.22%) and we were able to conclude that time- and frequency-domain features possessed the good discriminatory capability to classify normal and apneic events.

Some limitations can be observed in the study. Firstly, the SVM models were the part of the two-stage feature selection but not an automatic detection classifier, although they provided a better performance compared other studies mentioned in Section 1, such as in References [22,23]. In further work, various classification models will be conducted with the selected features in order to confirm which classifier emerges as the best. Secondly, the quality of bio-signals affects the performance of our method. Although removing artifacts and the baseline correction were used in this paper, it is necessary to upgrade the signal acquisition process and the sensor, such as wearable sensors that can be attached directly on human skin [37].

## 4. Conclusions

In conclusion, this paper aims to detect the feature subset with the highest discriminating power. Eighty-seven kinds of features were extracted using time-, frequency-domain, and non-linear algorithms from ECG, SaO_2_, airflow, abdominal, and thoracic signals. The *p*-values were computed by ANOVA and the rank-sum test, and the 87 extracted features were then classified into five classes (A, B, C, D, and E). The SVM was used to confirm the final feature subset, and *k*-fold cross-validation algorithm and the hill-climbing algorithm were both implemented. Class A (using 5 of the 87 features) illustrated the best performance, from ECG, SaO_2_, and abdominal signals. Also, the results showed that the Linear kernel with *R* = 1 outperformed the other classifiers. The proposed approach proves that a feature subset with high discriminative power can be useful in distinguishing apnea and normal sleep. Moreover, using the smaller subset reduces the complexity and the computation time.

## Figures and Tables

**Table 1:** Feature selection from PSG signals and their results (feature Nos. 1–62 from Electrocardiography (ECG) signals, Nos. 63–66 from SaO_2_ signals, Nos. 67–75 from airflow signals, Nos. 76–81 from abdominal signals and Nos. 82–87 from thoracic signals. Dashed lines divide the table according to the features of different signals).

Feature	λ_feature_	Feature	λ_feature_
1*.RMSSD_R_amp	3	44*.var_RR_0.03/0.4_	1
2*.NN50_RR	5	45*.spectral flatness	1
3*.SDSD_RR	3	46.spectral centroid	0
4*.tSD_RR	4	47*.spectral spread	5
5*.std_RR	4	48*.spectral decrease	3
6.mean	0	49*.spectral slope	2
7*.var	4	50*.V_MAX	3
8*.kurtosis	4	51*.DET	4
9*.mean_RR	4	52*.LAM	3
10*.CV_EDR	2	53.SCrC_1_RR	0
11*.mean_R_amp	1	54*.SCrC_2_RR	2
12*.var_EDR_D9	2	55*.SCrC_3_RR	2
13.var_EDR_D2	0	56*.SCrC_4_RR	4
14*.entropy_D1	3	57*.SCrC_5_RR	1
15*.entropy_D2	3	58*.std_PCA	1
16*.entropy_D3	3	59*.std_kPCA	1
17*.entropy_D4	3	60*.max_dia_PCA	4
18*.entropy_D5	3	61*.max_dia_kPCA	4
19*.entropy_D6	2	62*.RP_2_PC	2
3-4[0.8pt/2pt] 20*.entropy_D7	2	63*.med	4
21.entropy_A7	0	64*.RES4	5
22.mean_D1	0	65*.mean_PSD_0.016/0.05_	4
23.mean_D2	0	66*.SD_1_	5
3-4[0.8pt/2pt] 24.mean_D3	0	67*.mean	2
25.mean_D4	0	68*.med	3
26.mean_D5	0	69*.std	3
27*.mean_D6	1	70*.mean_PSD_0/0.1_	3
28.mean_D7	0	71*.mean_PSD_0.4/0.5_	2
29.mean_A7	0	72.mean_D1	0
30*.var_D1	3	73.mean_D2	0
31*.var_D2	4	74.mean_D3	0
32*.var_D3	4	75*.mean_A3	2
3-4[0.8pt/2pt] 33*.var_D4	3	76*.sum_abs	4
34*.var_D5	4	77*.std_abs	5
35*.var_D6	3	78*.mean	4
36*.var_D7	1	79*.mean_PSD_80/100_	2
37.var_A7	0	80.mean_D1	0
38*.WSD_RR	3	81.mean_D2	0
3-4[0.8pt/2pt] 39*.WSD_R_amp	4	82.sum	0
40*.max_PSD_0.03/0.5_	3	83*.std	3
41*.mean_PSD_10/20_	4	84*.med	4
42*.mean_PSD_80/100_	4	85.mean	0
43*.var_EDR_0.03/0.4_	1	86*.var	3
		87.mean_PSD_80/100_	0

**Table 2:** Feature classes obtained via the relationship between λ_feature_ and *ν*_PSG_.

Feature No.	Class A	Class B	Class C	Class D	Class E
	λ_feature_ = *ν*_PSG_	λ_feature_ = *ν*_PSG_-1	λ_feature_ = *ν*_PSG_-2	λ_feature_ = *ν*_PSG_-3	λ_feature_ = *ν*_PSG_-4
ECG	2 47	4 5 7 8 9 31 32 34 39 41 42 51 56 60 61	1 3 14 15 16 17 18 30 33 35 38 40 48 50 52	10 12 19 20 49 54 55 62	11 27 36 43 44 45 57 58 59
SaO_2_	64 66	63 65			
Airflow			68 69 70	67 71 75	
Abdominal	77	76 78		79	
Thoracic		83	84 86		

**Table 3:** Means (00.00%) of sensitivity specificity and accuracy of the *k*-fold method based on the feature classes using SVM models with different kernel functions and parameters (the worse accuracy (≤90%) is highlighted in bold).

Kernels	*R*	Class A			Class AB			Class ABC			Class A–D			Class A–E		
		Sen	Spe	Acc	Sen	Spe	Acc	Sen	Spe	Acc	Sen	Spe	Acc	Sen	Spe	Acc
RBF σ = 1	0.2	95.71	91.18	93.46	98.74	60.14	**79.42**	98.83	35.60	**67.07**	59.23	52.73	**54.44**	59.31	53.35	**55.58**
	1	95.52	94.65	95.07	98.56	80.44	**89.52**	98.77	62.59	**80.63**	98.75	41.54	**70.14**	98.84	27.24	**63.01**
	10	95.93	94.70	95.31	98.36	80.44	**89.37**	98.69	63.24	**80.97**	98.77	42.94	**70.85**	98.74	27.69	**63.18**
RBF σ = 5	0.2	92.97	92.75	92.86	96.85	95.92	96.39	96.75	96.16	96.46	97.28	93.50	95.39	97.75	92.60	95.16
	1	93.98	95.17	94.57	96.48	96.69	96.60	96.50	97.47	97.01	96.35	97.57	96.96	96.02	97.20	96.61
	10	94.08	95.99	95.05	96.47	96.83	96.64	96.10	97.47	96.77	96.43	97.01	96.70	96.04	97.37	96.71
RBF σ = 25	0.2	75.71	91.06	**83.40**	86.52	93.52	90.02	96.01	92.69	94.33	96.82	92.12	94.44	97.09	92.81	94.94
	1	87.20	89.49	**88.31**	91.60	93.96	92.78	96.70	94.65	95.67	96.91	94.62	95.76	96.83	94.54	95.68
	10	93.78	94.40	94.08	95.18	96.75	95.99	96.45	96.13	96.28	96.23	96.05	96.16	96.67	95.90	96.29
Poly *d* = 2	0.2	94.34	95.38	94.86	95.59	95.79	95.69	95.63	96.34	95.99	95.00	95.95	95.47	95.32	95.94	95.63
	1	94.48	95.24	94.85	93.87	95.91	94.89	95.57	95.73	95.68	94.80	95.24	95.00	95.70	96.73	96.20
	10	94.33	95.66	94.99	94.34	95.98	95.14	96.01	95.23	95.61	95.37	95.92	95.64	95.70	95.76	95.73
Poly *d* = 3	0.2	94.63	95.41	95.02	95.52	95.28	95.41	95.51	95.87	95.71	95.72	96.54	96.12	95.60	95.88	95.74
	1	94.71	94.86	94.79	94.85	95.46	95.14	95.96	96.80	96.33	95.71	96.08	95.89	96.86	80.61	**89.17**
	10	94.58	95.78	95.17	95.16	95.89	95.54	96.45	96.05	96.23	96.03	96.54	96.26	89.91	95.58	92.88
Poly *d* = 4	0.2	95.49	95.57	95.54	95.94	95.59	95.79	24.66	85.20	**54.96**	20.62	92.20	**56.32**	59.80	53.69	**55.79**
	1	94.97	95.87	95.39	95.72	96.30	96.01	05.97	97.61	**51.73**	20.76	85.14	**52.41**	21.48	91.12	**56.51**
	10	93.66	95.76	94.71	95.56	96.20	95.88	18.38	94.97	**56.74**	41.46	69.61	**55.49**	56.97	63.67	**60.32**
Linear	0.2	93.96	95.64	94.80	93.58	96.33	94.95	95.73	96.30	96.04	95.25	96.07	95.69	96.03	95.95	95.99
	1	94.29	96.17	95.22	93.90	96.03	94.95	95.86	96.16	96.01	95.96	96.44	96.20	95.75	96.38	96.07
	10	93.34	95.98	95.14	93.95	96.65	95.29	95.77	96.36	96.06	95.64	96.54	96.10	95.03	96.28	95.64

**Table 4:** Statistical performance (%) of means of sensitivity, specificity and accuracy in different feature classes.

Class	Sensitivity		Specificity		Accuracy	
	Mean	Std	Mean	Std	Mean	Std
A–E	87.21	19.72	82.68	22.47	84.89	16.50
A–D	84.71	24.89	86.68	17.84	85.59	16.72
ABC	85.06	28.22	89.45	15.55	87.25	15.24
AB	95.08	02.55	92.67	08.59	93.87	03.89
A	93.22	04.27	94.60	01.82	93.93	02.79

**Table 5:** Area of ROC (AUC %) on the *k*-fold using support vector machine (SVM) models with kernels and parameters (Class A features as input).

Kernels	*R*	AUC
RBF σ=1	0.2	93.44
	1	95.08
	10	95.31
RBF σ=5	0.2	92.86
	1	94.57
	10	95.03
RBF σ=25	0.2	83.38
	1	88.34
	10	94.09
Poly *d* = 2	0.2	94.86
	1	94.86
	10	94.99
Poly *d* = 3	0.2	95.02
	1	94.78
	10	95.18
Poly *d* = 4	0.2	95.53
	1	95.42
	10	94.71
Linear	0.2	94.80
	1	95.23
	10	94.66
